# Expression of the long non-coding RNA HOTAIR as a prognostic factor in squamous cell carcinoma of the head and neck: a systematic review and meta-analysis

**DOI:** 10.18632/oncotarget.20373

**Published:** 2017-08-21

**Authors:** Giuseppe Troiano, Vito Carlo Alberto Caponio, Linda Boldrup, Xiaolian Gu, Lorenzo Lo Muzio, Nicola Sgaramella, Lixiao Wang, Karin Nylander

**Affiliations:** ^1^ Department of Clinical and Experimental Medicine, University of Foggia, Foggia, Italy; ^2^ Department of Medical Biosciences/Pathology, Umeå University, Umeå, Sweden

**Keywords:** lncRNA, lncRNAs, non-coding RNA, HOTAIR, biomarker

## Abstract

**Introduction:**

Long noncoding RNAs (lncRNAs) are often dysregulated in cancer tissue and seem to play an important role in neoplastic processes. Recent studies have shown that the HOX transcript antisense intergenic RNA (HOTAIR) may play a role as a marker of prognosis in squamous cell carcinoma of the head and neck (SCCHN). The aim of this study was to perform a meta-analysis of studies focused on the prognostic role of HOTAIR in SCCHN.

**Results:**

At the end of the selection process, four studies were considered eligible for inclusion in the meta-analysis, comprising a total of 271 patients. Meta-analysis revealed that high expression of HOTAIR was associated with poor overall survival (HR, 1.90; 95% CI: [1.42, 2.53]; *p* < 0,0001), advanced tumor stage (OR, 3.44; 95% CI: [1.84, 6.43]; *p* < 0,001) and lymph-node metastasis (OR, 3.31; 95% CI: [1.24, 8.79]; *p* = 0,02).

**Materials and Methods:**

The literature search was performed in the following databases: PUBMED, SCOPUS, EMBASE and Web of Science, in order to find studies that met the inclusion criteria.

**Conclusions:**

Findings from this systematic review and meta-analysis revealed that HOTAIR represents a potential biomarker of prognosis in patients with squamous cell carcinoma of the head and neck.

## INTRODUCTION

Squamous cell carcinoma of the head and neck (SCCHN) encompasses a wide and frequent range of neoplasms arising from the epithelium of different sites such as: oral cavity, nasal cavity, oropharynx, hypopharynx and larynx [1, 2]. It has been reported that around 90% of all tumours in this region are squamous cell carcinomas [3]. SCCHNs are among the ten most frequent malignant neoplasms in humans; and due to the high rate of early metastasis to regional cervical lymph-nodes treatment of these patients is a challenge for the clinicians, [4]. It has been estimated that there will be > 300.000 new cases of lip and oral cavity SCC, > 140.000 of oropharyngeal SCC, > 85.000 of nasopharyngeal and > 155.000 of laryngeal SCC annually worldwide, with an increasing incidence and mortality rate [5]. Despite improvement in both surgical and adjuvant therapies, the 5-year survival rate for SCCHN has shown very little improvement over the last decades [6]. For such reasons, searching for biomarkers for diagnosis and prognosis represents a rising hope in order to provide clinicians with new tools for treatment of the disease.

Long non-coding RNAs (lncRNAs) are transcripts longer than 200 nucleotides previously defined as “junk DNA” [7]. They are usually transcribed by RNA polymerase II and can undergo splicing and polyadenylation processes. Although they are not translated into proteins, many of them are linked to some complexes involved in chromatin modification causing an overexpression or silencing of target genes of which most are in cancer pathogenesis [8].

The HOX transcript antisense intergenic RNA (HOTAIR) is one of the most studied lncRNAs [9, 10]. Different studies have focused on the capacity of HOTAIR to cooperate with different chromatin modifying complexes, above all the Polycomb Repressive Complex 2 (PRC2), through its 5′-terminal binding domain [11]. HOTAIR lets PRC2 recognize the target gene, leading to Histone H3 lysine-27 trimethylation, causing a silencing effect. HOTAIR is also able to cooperate with the LSD1 complex and can be regulated by different factors such as miR141, Ago2, c-Myc, TGF-β and small interfering RNAs (siRNAs) [12]. It is also involved in a regulatory control of p53 expression [13]. Different studies suggest that HOTAIR plays an important role in the metastatic process and may be a predictor of poor patient prognosis when highly expressed [14–17].

The aim of this systematic review and meta-analysis was to investigate the link between HOTAIR expression and patient prognosis in SCCHN in order to highlight its potential role as prognostic biomarker.

## MATERIALS AND METHODS

### Protocol and eligibility criteria

This systematic review and meta-analysis was performed according to the PRISMA (Preferred Reporting Items for Systematic Reviews and Meta-Analyses) guidelines [18] and the Cochrane Handbook [19]. This review has also been registered on the PROSPERO database (registration number: CRD42017057317), before the holding. Studies published in English and fulfilling the following criteria were considered eligible for inclusion in the study: (1) studies focusing on the expression of HOTAIR in SCCHN including more than 50 patients in total, (2) studies showing analysis of correlation between different levels of HOTAIR expression and overall survival, (3) studies reporting a Hazard Ratio (HR) and 95% confidence interval (CI) or Kaplan-Meier curve for HR estimation and (4) studies including quantitative analysis performed through quantitative PCR (qPCR), *in situ* hybridization (ISH), fluorescence *in situ* hybridization (FISH), droplet digital PCR (ddPCR) or RNA sequencing data. Accordingly, studies performed on cell lines or animal models, reviews, case reports, overlapping publications and all studies reporting insufficient data for estimation of HR and 95% CI, were excluded. No restriction was applied concerning year of publication.

### Information sources and search strategies

The literature search was performed in the following databases: PUBMED, SCOPUS, EMBASE and Web of Science, and independently performed by two of the authors (GT and CC). Mesh terms and free text words were combined through Boolean operators for research on databases. The following terms were used: (“long non-coding RNA” [Mesh] OR “lncRNA” [Mesh] OR “lincRNAs” [Mesh] OR “HOTAIR” [free word] OR “HOX transcript antisense RNA” [Mesh]) AND (“prognosis” [free word] OR “prognostic factor” [Mesh] OR “survival” [Mesh] OR “relapse” [Mesh]) AND ((“head neck” [free word] OR “oral cancer” [Mesh] OR “tongue” [Mesh] OR “pharynx” [Mesh] OR “HNSCC” [free word] OR “OSCC” [free word] OR “tonsil” [Mesh] OR “larynx” [Mesh])). In addition, a direct search on the bibliographies of previously published systematic reviews on the topic was also performed.

### Study selection, data collection process and data items

Eligibility assessment of the studies was performed independently in an unblinded standardized manner by two of the authors (GT and CC). In the first round title and abstract was read from the articles found, and studies meeting the inclusion criteria as well as those presenting insufficient data to make a clear decision were then read in full-text. Any disagreement was solved by discussion in a joint session. In order to investigate the level of agreement between the two reviewers, a value of K statistics was calculated. At the end of the selection process papers fulfilling all inclusion criteria were included in the quantitative synthesis. Data extraction was performed through an ad hoc extraction sheet and checked by two authors. In the quantitative synthesis a difference in overall survival was seen for patients with SCCHN showing different levels of HOTAIR expression. Furthermore, differences in: lymph-node metastasis, tumor stage and histological grade were seen between patients with Low or High HOTAIR expression.

### Risk of bias assessment, summary measures and planned methods for analyses

Analysis of risk of biases of the included studies was conducted using the Newcastle-Ottawa Scale (NOS) for case control studies. Risk of biases across studies was quantified evaluating the presence of heterogeneity among studies and also investigated through *Q* and *I*^2^ tests. A *p*-value of *Q* statistic < 0.05 was considered significant for presence of heterogeneity. Risk of biases across studies was quantified evaluating presence of heterogeneity among studies through *Q* and *I*^2^ tests. The Higgins index was also assessed and classified as follow: < 30% low heterogeneity, 30% to 60% medium heterogeneity, and > 60% high heterogeneity [20]. Overall effects were investigated with a fixed effect model where *I*^2^ was less than 50%, if higher, a random effects model was used. Data for overall survival were synthetized as HR and standard error (SE), while for lymph-node metastasis, tumor stage and histological grade the odds ratio (OR) was evaluated. In cases in which HR and its 95% CI was not reported in the article, it was extracted from Kaplan-Meier curves using the method of Tierney et al. [21]. All calculations were performed using Review Manager version 5.2.8 (Cochrane Collaboration, Copenhagen, 153 Denmark; 2014). Results of the meta-analysis were summarized in forest plots, and overall effects were compared using the inverse of variance test. A *p*-value lower than 0.05 was considered significant for all tests performed in this meta-analysis.

## RESULTS

### Study selection

**Figure 1:**
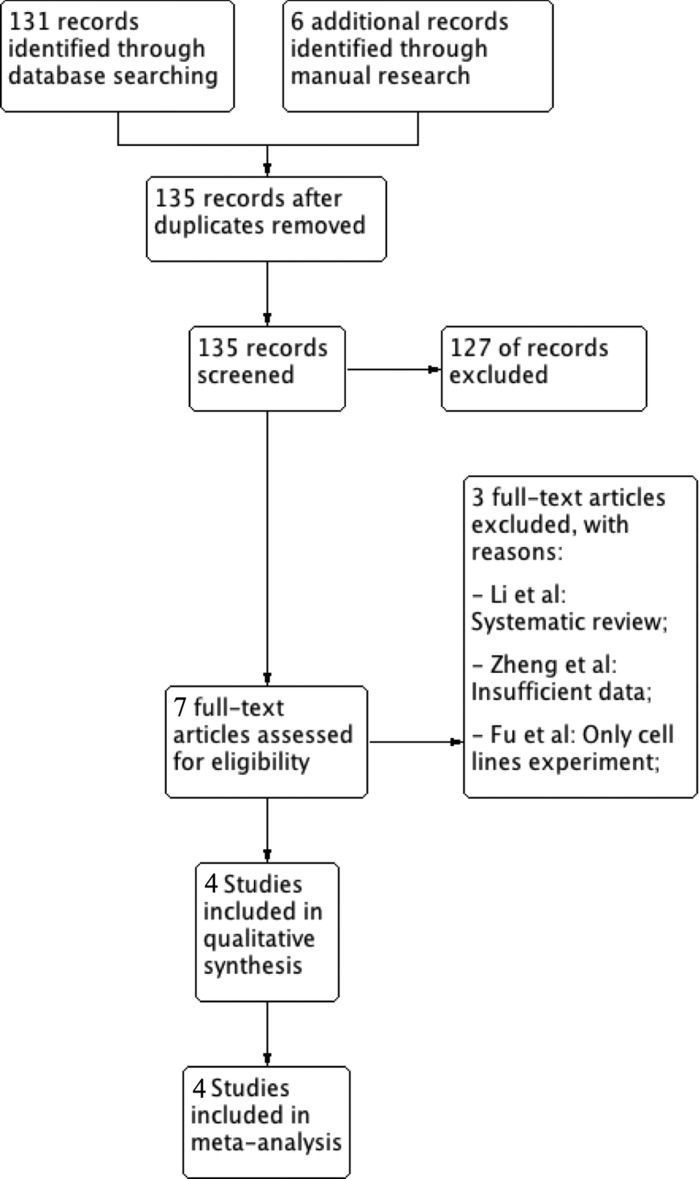
Flowchart for inclusion of studies in the meta-analysis and reasons for exclusion of articles read in full text

In order to evaluate inclusion in the review, a total of 135 records were screened by title and abstract. Among these only 7 were read full-text and at the end of the selection process 4 articles [22–25] were assessed as eligible for inclusion in the study. Three articles [26–28] were excluded. The reasons for exclusion and the flowchart of the selection process are reported in Figure 1. The value of k-statistic was 0.78 revealing an excellent level of agreement between reviewers.

### Study features and risk of bias within studies

In the 4 studies included in the meta-analysis [22–25], a total of 271 samples were analyzed, from tumors in different subsites of the head and neck area (Table 1). All studies were performed in China and published between 2012–2016. Two studies reported data for univariate analysis only [23, 24], while in the other two [22, 25] also multivariate analysis had been performed. In all studies quantification of HOTAIR expression was performed by the use of qPCR. In two studies GADPH was used as a single reference gene [23, 24], another study used 18 rRNA as internal control [25], while in the remaining study authors didn't specify the name of the reference gene used [22]. In addition, all studies reported a follow-up period of at least five years. Risk of bias assessment, performed with the Newcastle-Ottawa scale, showed that all included studies were at low risk of bias (Table 2).

**Table 1:** Features of the four studies included in the meta-analysis

Study	Year	Country	Involved Site	Total number	Detection method	Cut-off	Survival analysis	Multivariate analysis	Follow-up, months
Li	2012	China	Larynx	72	RT-qPCR	NA	OS	Yes	60
Wu	2015	China	Oral Cavity	50	RT-qPCR	Median	OS	No	60
Wu	2015	China	Tongue	76	RT-qPCR	NA	OS	No	100
Xu	2016	China	Head and Neck	73	RT-qPCR	NA	OS	Yes	150

**Table 2:** Summary of the risk of bias assessment using the Newcastle-Ottawa scale for case-control studies

Author/year	Country	Adequacy of case definition	Representativeness of cases	Controls Selection	Definition of controls	Comparability cases/controls	Ascertainment of exposure	Same method of ascertainment	Nonreponse rate
Li/2012	China	★	★	★	★	★★	★	★	NA
Wu/2015	China	★	★	★	★	★★	★	★	NA
Wu/2015	China	★	★	★	★	★★	★	★	NA
Xu/2016	China	★	★	★	★	★★	★	★	NA

### Synthesis of results and risk of bias across studies

**Figure 2:**
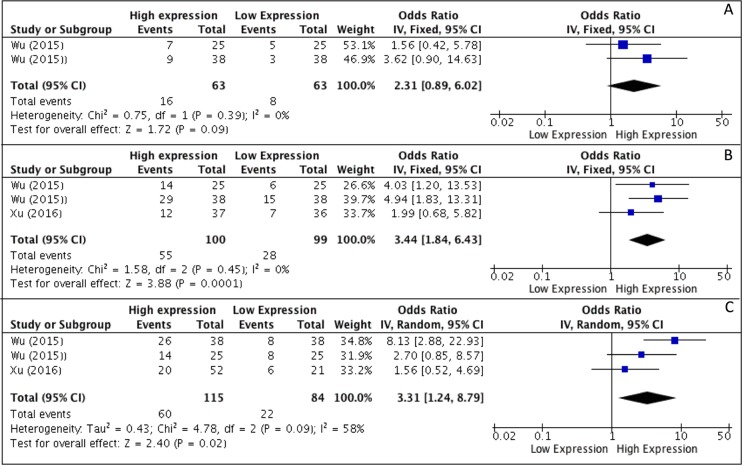
(**A**–**C**) Forest plot showing different expression of lncRNA HOTAIR and histological grade (A), tumor stage (B) and lymph-node metastasis (C); the frequency of HOTAIR expression was considered in patients with: Advance Stage (III–IV), positive LNM and Poor/Low Grade of differentiation.

**Figure 3:**
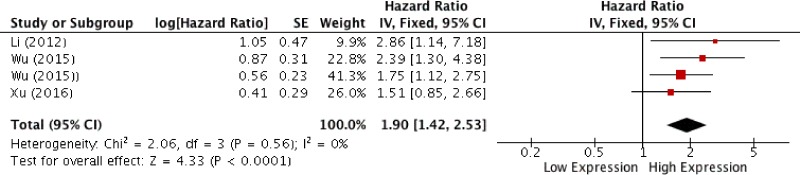
Forest plot for the association between HOTAIR expression and overall survival

Results of meta-analysis, on the basis of two studies, revealed that higher levels of HOTAIR expression were not associated with higher degree of differentiation *(OR, 2.31; 95% CI: [0.89, 6.02]; p = 0,09)* (Figure 2A), whereas a correlation to advanced tumor stages was seen *(OR, 3.44; 95% CI: [1.84, 6.43]; p < 0,001)* (Figure 2B). As heterogeneity was not detected *(I*^2^ *= 0%)* a fixed effects model was used. Analysis of correlations performed with a random effect model *(I*^2^ *= 58%)* revealed a correlation between HOTAIR expression and the rate of LNM *(OR, 3.31; 95% CI: [1.24, 8.79]; p = 0,02)* (Figure 2C). Analysis of overall survival pooling HRs showed high expression of HOTAIR to be associated with poor OS in patients with SCCHN *(HR, 1.90; 95% CI: [1.42, 2.53]; p < 0,0001)*, (Figure 3). Summary of data extracted from the included studies used for the pooled analysis are shown in Table 3.

**Table 3:** Synthesis of data extracted from the included studies related to outcomes pooled in the meta-analysis

HOTAIR expression										
Study	High with LNM	Low with LNM	High with high grade	Low with high grade	High with high staging	Low with high staging	High expression	Low expression	HR statistic	Hazard ratios (95% CI)
Li	NA	NA	NA	NA	NA	NA	33	39	Survival curve and data in paper	2,41–2,856
Wu	14	8	7	5	14	6	25	25	Survival curve	2.3
Wu	26	8	9	3	29	15	38	38	Survival curve	2.39
Xu	20	6	NA	NA	12	7	52	21	Survival curve	1.51
**Total**	**136**	**80**	**24**	**11**	**106**	**54**	**239**	**192**		

## DISCUSSION

Non-coding RNAs (ncRNAs) are a class of transcripts involved in various cellular processes [29], and classified into three major groups: short ncRNAs (20–50 nt long), medium ncRNAs (50–200 nt long) and long ncRNAs (> 200 nt long). Long non-coding RNAs (lncRNAs) are widely transcribed in the genome and their expression seems to be dysregulated in many diseases [30–32]. Dysregulation of the expression of lncRNAs has been seen in different cancer types, suggesting that these molecules could be involved in tumorigenesis [33–35]. Due to results indicating an important role in cancer, HOTAIR is one of the most studied lncRNAs [11]. HOTAIR is 2.2 kb long and transcribed from the antisense strand of the *HOXC* gene located on chromosome 2, and consisting of 6 exons [36, 37]. After interaction with the polycomb repressive complex 2 (PRC2) and lysine-specific histone demethylase 1 (LSD1) It is able to regulate gene expression through chromatin dynamics [38, 39]. A recently published study, however, showed that HOTAIR can lead to transcriptional changes independent of PRC2 activity [40]. Furthermore, HOTAIR can act as a competitive endogenous RNA (ceRNA) and regulate levels of some microRNAs [41], for example it can decrease expression of miR-331–3p leading to upregulation of the human epithelial growth factor 2 (HER2), a well-known oncogene [42]. In SCCHN, HOTAIR is able to regulate the activity of the well-known oncogene PTEN, by influencing promoter methylation [25]. In addition, it plays an important role in the epithelial-mesenchymal transition (EMT) repressing expression of E-cadherin through interaction with EZH2 [23, 27]. Overexpression of HOTAIR can also inhibit apoptosis through modification of the mitochondrial membrane and activation of the mitochondrial calcium uptake dependent cell death [43]. It also plays a role in progression and metastasis through a regulatory loop with HuR. In addition, it can work as ceRNA for miR-7 thus avoiding its inhibitory effect on HuR expression [22].

Previous meta-analyses have demonstrated higher expression of HOTAIR to correlate with poor prognosis in patients with cervical [44], ovarian [45], esophageal [44] and gastric [46] cancer. However, to our knowledge no meta-analysis has evaluated its role as a prognostic predictor in SCCHN. In this meta-analysis four studies evaluating correlations between clinical, pathological and survival parameters were included, and a total of 271 patients analyzed. Pooled analysis showed reliable evidence for higher levels of HOTAIR expression to correlate with poor prognosis in SCCHN (HR, 1.90; 95% CI: [1.42, 2.53]; *p* < 0,0001). In addition, meta-analysis demonstrated a correlation between higher levels of HOTAIR and higher rates of LNM (OR, 3.31; 95% CI: [1.24, 8.79]; *p* = 0,02). However, presence of heterogeneity *(I^2^= 58%)* limits the reliability of these findings, it could be speculated that increasing the number and power of the included studies could render more reliable results. Very interestingly, increased levels of HOTAIR were also associated with advanced tumor stage (OR, 3.44; 95% CI: [1.84, 6.43]; *p* < 0,001) in the absence of heterogeneity *(I^2^ = 0%)*. Looking at results for histological degree of differentiation, meta-analysis failed to demonstrate a correlation with HOTAIR expression (OR, 2.31; 95% CI: [0.89, 6.02] *p* = 0.09). However, only two studies were pooled in the evaluation of this outcome, resulting in very low power of evidences.

Taken together, the present results suggest a potentially important role for HOTAIR as a biomarker of aggressiveness in SCCHN. Results of this meta-analysis should, however, be read with caution due to some obvious limitations. First of all, the number of studies included was low. Secondly a geographical bias may be present as all studies were performed in China, rendering data only from one ethnic group, which could influence the validity of the results. As demonstrated in previous studies, people of different race/ethnicity vary in their risk of developing SCCHN [47, 48]. In addition, differences in prognosis have been seen for patients of different ethnicities, linked both to the genetic and the lifestyle variabilities [49]. As it is known that the frequency of smoking or chewing tobacco, alcohol consumption and HPV infection differs among populations, there can be a “geographical bias” between studies [48, 50]. In addition, the studies included in the meta-analysis have evaluated samples from different subsites within the head and neck area, sites that can differ in both clinical and molecular behavior [51–53]. Furthermore, not all studies reported time-to-event outcome with a multivariate approach, and cut-off values differed and were, in some of the included articles, not even reported. Multivariate analysis allows adjustment for patient-related factors, known as covariates or confounders, which could potentially affect the survival time of the patients [54, 55]. Whether for example a higher number of patients with advanced stage is present in a group, the difference in prognosis between groups could be influenced by the Staging distribution and not HOTAIR expression. On the other hand, the low rate of heterogeneity recorded in three of the studies, combined with a follow-up time of at least 60 months enhances the reliability of findings.

In conclusion, findings from this meta-analysis indicate that higher expression of lncRNA HOTAIR is associated with poor prognosis, advanced tumor stage and higher rate of lymph-node metastasis.

Based on the present results, quantification of HOTAIR could be recommended as a complement associated with evaluation of other prognostical, clinical and molecular biomarkers, in order to categorize patients with high risk of death and thus to help clinicians in the choice of best personalized treatment option. Furthermore, this study encourages the execution of large and well-standardized multicenter studies on human samples of different ethnicity in order to confirm these preliminary findings.
